# Contribution of socioeconomic status, stature and birth weight to obesity in Sub-Saharan Africa: cross-sectional data from primary school-age children in Cameroon

**DOI:** 10.1186/1471-2458-14-320

**Published:** 2014-04-07

**Authors:** Lifoter K Navti, Uta Ferrari, Emmanuel Tange, Susanne Bechtold-Dalla Pozza, Klaus G Parhofer

**Affiliations:** 1 CIHLMU Center for International Health at Ludwig-Maximilians-Universitaet, Munich, Germany; 2 Department of Biochemistry, Catholic University of Cameroon (CATUC), P.O. Box 782, Bamenda, Cameroon; 3 Diabetes Research Group, Department of Medicine IV, Ludwig-Maximilians-Universitaet, Ziemssenstr. 1, Munich 80336, Germany; 4 Department of Food Science and Technology, Catholic University of Cameroon (CATUC), P.O. Box 782, Bamenda, Cameroon; 5 Pediatric Endocrinology and Diabetology, University Children’s Hospital, Ludwig-Maximilians-Universitaet, Lindwurmstr. 4, Munich 80337, Germany; 6 Department of Medicine II - Grosshadern, Ludwig-Maximilians-Universitaet, Marchioninistr. 15, Munich 81377, Germany

**Keywords:** Socioeconomic status, Obesity, Height, Children

## Abstract

**Background:**

The pattern of obesity in relation to socioeconomic status is of public health concern. This study investigates whether the association between height and obesity in children is affected by their socioeconomic background. It also explores the relationship between high birth weight and obesity.

**Methods:**

School children, (N = 557; 5 to 12 years old) were recruited from randomly selected primary schools in a cross-sectional study including 173 rural and 384 urban children in the North West Region of Cameroon. Socioeconomic status (SES) and birth weight were obtained using a self administered questionnaire. Anthropometric measures included height, weight, BMI, waist circumference and percentage body fat. These measures were transformed into age and sex-standardized variables. Then participants were divided according to quartiles of height SDS.

**Results:**

The highest frequencies of overweight/obesity (18.8%), abdominal overweight/obesity (10.9%) and high body fat/obesity (12.3%) were observed among the tallest children from a high socioeconomic background. Univariate analyses indicate that children of high SES (39.9%), fourth height quartile (33.1%) and of high birth weight (54.8%) were significantly (*p* < 0.001) more likely to be overweight/obese. Multivariate analyses showed high SES (OR 8.3, 95% CI 3.9 – 15.4), fourth height quartile (OR 9.1, 95% CI 3.4 – 16.7) and high birth weight (OR 0.1, 95% CI 0.06 – 0.2) as independent predictors of overweight/obesity.

**Conclusions:**

This study confirms that children coming from a high socioeconomic background and being tall are at particular risk of becoming obese.

## Background

The escalating numbers of children affected with overweight and obesity remains a global challenge [1]. This has continued to be a mounting concern among researchers as studies using different obesity parameters have shown that obesity increases the risk of adverse health outcomes including cardiovascular diseases [2], certain cancers, psychological problems and diabetes [3]. Many studies have identified different factors that could explain to various extents the current dramatic global increase in the prevalence of overweight and obesity, which include: decreased physical activity [4], food marketing practices [5], genetic influence [6,7], maternal smoking during pregnancy [8], high birth weight [9], increased television viewing [10], higher dietary energy density [11], parental obesity [12] and more. Additionally, a review had indicated the contribution of socioeconomic background to social inequalities in the distribution of the frequency of obesity [13]. This indicates that overweight and obesity involves a complex interaction between many variables in childhood, adulthood and old age. The majority of the studies above have been carried out in developed countries.

Sub-Saharan African countries including Cameroon are at various stages of economic development. Because of the increased attention gained by social inequalities in health, the close monitoring of the socioeconomic pattern of obesity especially among school children [14] in this region is important. There has been little attention to this group of children and if nothing is done, this might pose a major human and economic cost to this region in the future and might affect the sustainability of the healthcare systems, which are already over burdened with high rates of infectious diseases.

In developing economies, overweight and obesity tends to affect more people from a high socioeconomic background [14,15]. In contrast, in developed nations obesity is inversely related to socioeconomic status (SES), a situation which is more pronounced in women [16]. This inverse relationship has also been confirmed in children [17,18], with parental social class contributing to offspring obesity [19].

There is evidence indicating that children from a low income group were significantly shorter than those from a high income group and regardless of the income background, overweight and obese children were taller than their non-overweight and non-obese peers [20]. Even though it is documented that increased height among children is associated with increased risk of obesity [21-23], evidence from Chile during the nutrition transition shows that both tallness and stunting in children are associated to obesity [24].

High birth weight has also been documented as an indicator for later obesity. For instance, some studies [9,25,26] have shown a positive association between high birth weight and obesity later in life. However, a cohort study did not find any association between birth weight and adult body mass index [27].

The aim of this study is to explore the interactions between SES, height and obesity and test the hypothesis that the height-obesity association is greater in school-age children from a high socioeconomic background. It also explores the relationship between high birth weight and overweight/obesity.

## Methods

### Subjects

The data used in this study were collected cross-sectionally between February and June 2012, from children living and attending primary education in the North West Region of Cameroon including a mix of socioeconomic groups from rural and urban populations. The analyses consisted of 557 participants (173 from rural and 384 from urban settings) of ages 5 to 12 years and were recruited from both private and public schools selected at random. Consent information, which explained the purpose of the study, was distributed in the schools to parents or guardians and the head teachers accompanied by the study questionnaire. Also, the principal investigator (L.K.N) had the opportunity to explain the aims of the study to parents or guardians during the Parents Teachers Association (PTA) meetings, which usually take place within the academic year. In return a signed informed consent certificate was obtained from each parent or guardian and school head teacher before measurements were carried out. Dates of birth and gender were collected from school records at the same time as the anthropometric measurements.

### Anthropometry

All measurements were school-based and conducted by well trained nurses ensuring that standard protocols were respected. Assent was obtained from each participant before measurements. Standing height was measured close to 0.1 cm without shoes using a portable stadiometer (Seca 213, Germany). A digital scale (Omron BF 511, Japan) was used to measure body weight to the nearest 0.1 kg with children dressed in light school uniforms and shoes taken off. Then height and weight were used to calculate body mass index (kg/m^2^) as a general measure of weight status.

Waist circumference, a measure of abdominal fatness, was measured as recommended by McCarthy *et al.*[28] using a non-elastic flexible tape (Seca 201, Germany).

The formula of Deurenberg *et al.* was used to estimate percentage body fat in children. %BF = 1.51 × BMI – 0.70 × age – 3.6 × sex + 1.4 (where males = 1, females = 0). This formula has been validated elsewhere [29].

### Socioeconomic status

The Cameroon classification system of occupation and income; civil servants categories C, B and A of the public service, was used to categorize participants into low, middle and high socioeconomic status (SES) respectively [15]. This information was obtained from parents or guardians whose children participated in the study using a structured questionnaire designed in English and French. The highest level of SES of either parent was used to assign each child to the appropriate category of SES. Parental level of education was also assessed using the questionnaire and four categories were established: illiterate (attended no school), primary (1 – 6 years of education), secondary (7 – 13 years of education) and higher education (>13 years of education). The categorization of participants was based on the parent with the highest level of education. SES levels were obtained from 522 out of the 557 parents giving a response rate of 93.7%. Also, a response rate of 89.05% was obtained for parental level of education.

### Birth weight

Birth weight was self reported by parents on the questionnaire and in the analysis high birth weight was indicated as birth weight > 4 kg. These readings were obtained from 454 parents giving a response rate of 81.5%.

### Ethical and administrative clearances

Approval for this study was obtained from the Institutional Review Board of the Biotechnology Center of the University of Yaoundé I. Administrative clearances were obtained from the Regional Delegation for Public Health and the Regional Delegation for Basic Education of the North West Region of Cameroon. All parents gave written informed consent before any study related procedure was performed.

### Statistical analysis

SPSS for Windows version 16.0 was used for analyses. Height, weight and BMI were converted to their corresponding standard deviation scores (SDS) using the WHO growth reference data [30]. Also, waist circumference and percentage body fat were standardized for age and sex using the UK reference data for waist circumference [28] and percentage body fat [31]. The study participants were sorted according to increasing height SDS and then divided into quartiles of height SDS. The mean height and weight SDSs were also compared across the socioeconomic groups using a 1-way ANOVA with post hoc Bonferroni test. A parametric *t* test was used to compare means of age-adjusted and unadjusted anthropometric measures between boys and girls.

Overweight and obesity were defined using the WHO cut-off points [30], while the 91st percentile [28] was used to define abdominal overweight/obesity and the 85th percentile [31] used for high body fat/obesity.

In addition, the frequencies of overweight/obesity, abdominal overweight/obesity and high body fat/obesity stratified by SES, quartiles of height SDS, and high birth weight (determinants) were calculated. This was followed by univariate analysis, which was done using binary logistic regression models to estimate the corresponding odds ratios (OR) (adjusted for age and gender) with 95% CI and *p*-values. Further, multivariate binary logistic regression analysis was performed to determine the independent association between each of the determinants and the obesity measures. SES, height SDS and birth weight showed significant associations with obesity in the univariate analysis and were included in the model as well as age and gender during the multivariate analysis.

Moreover, height SDS showed significant interactions with SES in this study. Therefore, the frequencies of overweight/obesity, abdominal overweight/obesity and high body fat/obesity according to quartiles of height SDS differentiated by the socioeconomic groups were estimated. A *p*-value of 0.05 was used to indicate statistical significance.

## Results

There were significant differences (*p* < 0.05) in BMI SDS, WC SDS and %BF between boys and girls in this study. Table 1 shows the main characteristics of the study population (N = 557), 48.5% were female and 51.5% were male. 31.1% of the subjects were from the rural areas whereas 68.9% from urban areas. Less than 30% of the participants were from the middle or high socioeconomic background. More girls than boys were categorized as overweight or obese on the basis of waist circumference or percentage body fat.

**Table 1:** **Descriptive characteristics by gender, n [% (95**% **CI)], mean age 9.0 ± 1.8 years**

**Variables**	**Girls**			**Boys**		
	**N = 270**			**N = 287**		
	**n**	**[% (95% CI)]**		**n**	**[% (95% CI)]**	
Degree of urbanization						
Rural	83	30.7	(25.5 - 36.5)	90	31.4	(26.2 - 36.9)
Urban	187	69.3	(61.3 - 76.7)	197	68.6	(63.0 - 73.8)
Socioeconomic status						
Low	117	43.3	(37.6 - 49.2)	123	42.9	(37.3 - 48.7)
Middle	70	25.9	(21.0 - 31.5)	74	25.8	(21.1 - 31.1)
High	73	27.0	(22.1 - 32.6)	65	22.6	(18.2 - 27.8)
Missing data	10	3.7	-	25	8.7	-
Body mass index^a^						
Thinness	5	1.9	(0.7 - 4.4)	1	0.3	(0.0 - 1.9)
Normal	219	81.1	(75.6 - 85.0)	235	81.9	(77.0 - 85.9)
Overweight and obesity	46	17.0	(12.9 - 22.0)	51	17.8	(12.1 - 24.7)
Waist circumference^b^						
Small	2	0.7	(0.2 - 2.7)	3	1.0	(0.3 - 3.1)
Normal	220	81.5	(74.8 - 87.0)	256	89.2	(80.2 - 98.5)
Abdominal overweight and obesity	48	17.8	(11.8 - 25.1)	28	9.8	(7.1 - 14.0)
Percentage body fat^b^						
Low	8	3.0	(1.5 - 5.7)	3	1.0	(0.4 - 3.0)
Normal	220	81.5	(76.4 - 85.7)	268	93.4	(89.9 - 95.7)
High body fat and obesity	42	15.5	(11.7 - 20.4)	16	5.6	(3.5 - 8.9)

^ab^relative to WHO 2007, UK 1990 growth reference data respectively.

Also, Table 2 shows that obesity was more pronounced among urban girls than boys when all three parameters of obesity were used.

**Table 2:** Prevalence of excess body fat in relation to degree of urbanization

	**Gender**	**Urban**		**Rural**	
		**%**	**(95% CI)**	**%**	**(95% CI)**
Overweight/obesity (BMI)	Boys	17.8	(13.1-23.7)	17.7	(11.3-26.9)
	Girls	18.2	(13.3-24.3)	14.5	(8.5-23.6)
Abdominal overweight/obesity (WC)	Boys	10.2	(6.5-15.2)	8.9	(4.6-16.6)
	Girls	20.9	(15.7-27.2)	10.8	(5.8-19.3)
High body fat/obesity (%BF)	Boys	6.6	(3.9-11.0)	3.3	(1.1-9.4)
	Girls	17.6	(12.9-23.8)	10.8	(5.8-19.3)

CI: confidence interval

Univariate analysis in Table 3 show that regardless of which measure of obesity used, a high socioeconomic background, tallness and a high birth weight were significantly associated with a higher frequency of overweight/obesity, abdominal overweight/obesity and high body fat/obesity after adjusting for age and gender. This association was more pronounced when BMI is used as a measure of obesity. However, parental level of education did not show any significant association with the parameters of obesity in univariate analysis.

**Table 3:** **Frequency and OR (95**% **CI) for the association of excess body fat with potential determinants**

**Determinants**	**N 557**	**Overweight/obesity (BMI)**			**Abdominal overweight/obesity (WC)**			**High body/obesity (%BF)**		
		**Frequency (%)**^**a**^	**OR (95% CI)**	***p*-value**	**Frequency (%)**^**b**^	**OR (95% CI)**	***p*-value**	**Frequency (%)**^**c**^	**OR (95% CI)**	***p*-value**
Socioeconomic status										
High	138	39.9	10.1 (5.4 - 18.9)	< 0.001	26.1	3.5 (1.9 - 6.2)	< 0.001	23.2	0.2 (0.1 - 0.5)	< 0.001
Medium	144	16.0	3.5 (2.0 - 6.2)	< 0.001	11.1	2.8 (1.5 - 5.3)	0.002	9.0	0.1 (0.05 - 0.3)	< 0.001
Low	240	6.3	ref.		9.2	ref.		4.6	ref.	
Missing data	35									
Quartiles of height SDS										
Fourth quartile	139	33.1	10.9 (4.6 - 25.8)	< 0.001	30.2	4.2 (2.2 - 8.2)	< 0.001	23.7	0.3 (0.1 - 0.7)	0.006
Third quartile	137	20.4	2.3 (1.3 - 4.0)	0.004	12.4	3.8 (2.0 - 7.2)	< 0.001	8.0	0.3 (0.1 - 0.7)	0.007
Second	141	19.1	3.6 (1.9 - 6.6)	< 0.001	11.3	ref.		7.8	0.1 (0.03 - 0.4)	< 0.001
First	140	5.0	ref.		0.7			2.1	ref.	
High birth weight (>4 kg)										
Yes	95	54.8	8.7 (5.2 - 14.5)	< 0.001	34.7	5.2 (2.9 - 9.1)	< 0.001	27.4	7.1 (3.5 - 14.7)	< 0.001
No	359	12.3	ref.		9.7	ref.		7.0	ref.	
Missing data	103									

^abc^Based on WHO 2007 BMI, UK 1990 91^st^ centile and UK 1990 85^th^ centile cut-offs respectively. Abbreviations: OR. Odds ratio; CI, confidence interval. (odds ratios adjusted for age and gender). Overweight/obesity, abdominal overweight/obesity and high body fat/obesity are defined by BMI, waist circumference and% body fat respectively.

The results of the multivariate analysis are shown on Table 4. The model showed a statistically significant independent association between overweight/obesity, abdominal overweight/obesity and high body fat/obesity and SES, height SDS and high birth weight.

**Table 4:** Multivariate binary logistic regression analysis with overweight/obesity (BMI), abdominal overweight/obesity (WC) and high body fat/obesity (%BF) as dependent variable

	**Overweight/obesity (BMI)**		**Abdominal overweight/obesity (WC)**		**High body fat/obesity (%BF)**	
	**OR (95% CI)**	***p*-value**	**OR (95% CI)**	***p*-value**	**OR (95% CI)**	***p*-value**
Socioeconomic status						
High	8.3 (3.9 - 15.4)	< 0.001	2.5 (1.2 - 5.6)	0.017	0.2 (0.1 - 0.6)	0.003
Medium	3.7 (1.8 - 7.3)	< 0.001	2.1 (1.8 - 7.3)	< 0.001	0.1 (0.05 - 0.4)	< 0.001
Low	ref.		ref.		ref.	
Quartiles of height SDS						
Fourth quartile	9.1 (3.4 - 16.7)	< 0.001	4.1 (1.9 - 8.7)	< 0.001	0.3 (0.1 - 0.7)	0.010
Third quartile	2.1 (1.0 - 4.1)	0.041	3.8 (1.8 - 8.1)	< 0.001	0.4 (0.2 - 0.9)	0.041
Second	3.7 (1.7 - 7.9)	0.001	ref.		0.2 (0.05 - 0.7)	0.014
First	ref.				ref.	
High birth weight (>4 kg)						
Yes	0.1 (0.06 - 0.2)	< 0.001	0.2 (0.1 - 0.4)	< 0.001	7.1 (3.2 - 15.9)	< 0.001
No	ref.		ref.		ref.	

OR, odds ratio; CI, confidence interval; (odds ratios adjusted for age and gender). Overweight/obesity, abdominal overweight/obesity and high body fat/obesity are defined by BMI, waist circumference and% body fat respectively.

**Figure 1:**
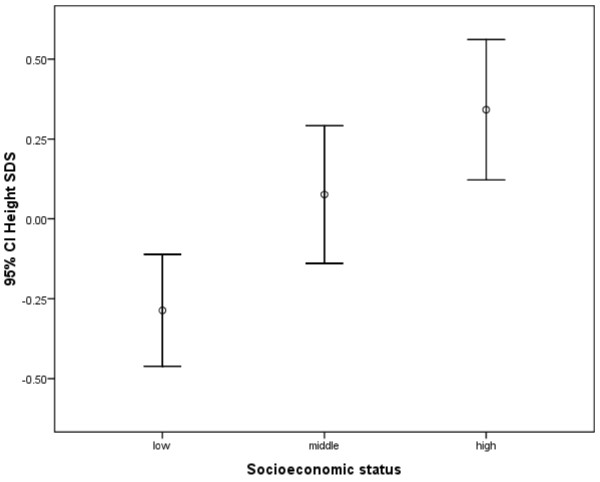
**Height differences by socioeconomic status.** Significant difference observed between low and high SES (*p* < 0.001). N = 522 after exclusion of subjects with no information on SES.

Figure 1 shows that participants from the high socioeconomic background were significantly (*p* < 0.001) taller than those from the low socioeconomic group, which corresponds to a 0.6 unit difference in height SDS. However, there were no significant differences in height SDS between the low and middle SES groups and also between the middle and high SES groups after carrying out a 1-way ANOVA with post hoc Bonferroni test.

Based on the significant difference in height observed between the low and high SES group, Table 5 shows the frequency of excess body fat tabulated in the three socioeconomic groups and quartiles of height SDS. It indicates that the frequencies of overweight/obesity, abdominal overweight/obesity and high body fat/obesity were highest among the tallest children from the high socioeconomic group. Also, in the second, third and fourth quartiles of height SDS, there was a progressive increase in the frequency of obesity with increasing SES. In this study, weight SDS also differed significantly within the socioeconomic groups.

**Table 5:** Frequency of excess body fat according to quartiles of height SDS differentiated by socioeconomic status

**Quartiles of height SDS**	**Socioeconomic status**					
	**Low**		**Middle**		**High**	
	**%**	**(95% CI)**	**%**	**(95% CI)**	**%**	**(95% CI)**
Overweight/obesity (BMI)						
First quartile	1.3	(0.4 - 3.6)	2.1	(0.7 - 6.0)	0.7	(0.1 - 4.0)
Second quartile	1.7	(0.7 - 4.2)	4.2	(1.9 - 8.9)	10.1	(6.1 - 16.3)
Third quartile	0.8	(0.2 - 3.0)	1.4	(0.4 - 4.9)	10.1	(6.1 - 16.3)
Fourth quartile	2.5	(1.2 - 5.4)	7.6	(4.3 - 13.2)	18.8	(13.2 - 26.2)
Abdominal overweight/obesity (WC)						
First quartile	0.0	(0.0 - 1.6)	0.7	(0.1 - 3.8)	0.0	(0.0 - 2.7)
Second quartile	1.3	(0.4 - 3.6)	1.4	(0.4 - 4.9)	7.3	(4.0 - 12.8)
Third quartile	1.3	(0.4 - 3.6)	2.1	(0.7 - 6.0)	8.0	(4.5 - 13.7)
Fourth quartile	6.7	(4.1 - 10.6)	6.9	(3.8-12.3)	10.9	(6.7 - 17.2)
High body fat/obesity (%BF)						
First quartile	0.8	(0.2 - 3.0)	0.0	(0.0 - 2.6)	0.7	(0.1 - 4.0)
Second quartile	1.3	(0.4 - 3.6)	1.4	(0.4 - 4.9)	4.4	(2.0 - 9.2)
Third quartile	0.0	(0.0 - 1.6)	2.1	(0.7 - 6.0)	5.8	(3.0 - 11.0)
Fourth quartile	2.5	(1.2 - 5.4)	5.6	(2.8 - 10.6)	12.3	(7.8 - 18.8)

N = 522 after exclusion of subjects with no information on SES; CI, confidence interval.

## Discussion

This study set out firstly to examine the effect of socioeconomic background on the relationship between height SDS and three measures of obesity, and secondly to determine the relationship between high birth weight and obesity in children.

It was demonstrated that the frequency of excess body fat was highest among the tallest children in the high socioeconomic group. Also, the study confirmed from the multivariate analyses that high SES, tallness, and high birth weight were strong independent predictors of obesity. This was consistent for all three parameters of obesity.

A recent study among adults in urban Cameroon showed a positive association between obesity and SES [15]. This has been attributed to the current nutrition transition experienced in Sub-Saharan countries. As the economies and peoples’ earnings are getting better, they tend to adopt Western lifestyles decreasing their physical activity levels [32,33]. A shift in dietary pattern from fruits, vegetables and grains to an increased intake of animal fats, sugar and salt as a result of better income has also been attributed to the rise in obesity levels in developing countries [5]. These lifestyle changes are reflected in Cameroon children and could explain why some of them accumulate more body fat.

Another study in urban Cameroon had indicated that the prevalence of overweight was high among adolescents from all socioeconomic groups; the low (8%), middle (11%) and high (9%), with girls more likely to be overweight than boys [34]. Our study has also found a higher frequency of excess body fat among urban girls. In addition to the latter, our study has demonstrated the existence of a much more pronounced SES gradient in the distribution of the prevalence of excess body fat in school age children. More children from the high socioeconomic background are affected than their peers from the low socioeconomic group. Even though there is no documented evidence, in the past, children used to walk to school, an activity that burnt lots of calories in children. However, motorized transportation to schools nowadays, a common phenomenon in urban settings, which is easily affordable by parents of a higher socioeconomic status, could contribute to some extent in lowering physical activity levels in children. Also children now spend more hours in sedentary activities like television watching, which has been associated to overweight and obesity in developed countries [35-37]. However, in relation to income group, studies in the developed world have shown that overweight and obesity affects mostly people from disadvantaged groups [18,20,38].

Children from the high SES group were significantly taller than those from the low SES group. A recent study made a similar observation and described this situation to be ‘largely associated to a relative height-growth restriction’ [20]. However, in our study, age and sex-adjusted weight was also significantly different in the socioeconomic groups, meaning that overall weight could also have contributed to the high prevalence of excess body fat among children from the high SES group.

The high prevalence of obesity among subjects from a high SES could also be explained by the fact that food preparation patterns have changed over time with homemade foods gradually being replaced by more convenient and high calorie ready to eat foods [5] especially in urban areas. Also, historically, children used to take home made lunch to school. Nowadays, high income earners prefer to give pocket allowances to children who will spend it on high sugar containing snacks sold near school premises. In addition, children are a target to the advertisement of fizzy drinks and confectioneries [5], which are easily afforded by the rich parents in developing economies. No dietary information was obtained from the children in this study to substantiate the above points. In fact, a study indicated the potential of poor nutrition and decreased physical activity to induce obesity and linear growth in children [20].

Our study has shown a significant relation between height SDS and obesity. Similar findings have been indicated in other studies [22,23]. Possible reasons for this relationship include a hormonal imbalance between insulin-like growth factor 1 and growth hormone [39] and early high protein intake [40]. The source of protein is still a subject of debate. High birth weight was also associated to obesity in both univariate and multivariate analyses. A review indicated that high birth weight is associated to higher obesity levels in adulthood [41].

This study did not find any association between parental level of education and the different parameters of obesity in the children. Similarly, a study in urban Cameroon did not find any significant association between obesity and level of education in adults [15]. The authors indicated that this reflects the Cameroon society of today where a better education is not necessarily associated to a higher salary. Those involved in financially rewarding economic activities are often the less educated [15].

This study had limitations worth mentioning. Birth weight was not available for all participants and bias could have been possible as birth weight was self reported by parents. Also, the tanner stages of children were not estimated and early puberty could have affected the height-obesity relationship especially among girls who had higher WC and %BF readings. This study made use of UK references for WC and percentage body fat and it is unclear if these references can be applied to our study population. Percentage body fat was predicted from an equation that was validated in a study in Europe and it is not certain if this can be applied to this study. Further, the influence of genetics on height cannot be completely ruled out. This study used parental occupation, income and level of education as indicators of the SES of participants. However, a study in urban Cameroon has shown the World Bank household amenities score as a better indicator for SES in developing economies [15]. Information on SES was not available for all subjects in this study. It is possible that some of the parents considered this as confidential information or were unwilling to confide as to what may happen with this information [15]. This study used the Cameroon public service classification system which does not adequately reflect the socioeconomic background of those in the private sector as well as housewives at any one moment. Finally, the limited number of children with excess body fat in this study did not permit us to examine if the associations differed by sex.

Despite the above limitations, this study has provided data for the first time for Cameroon to examine the height-obesity-SES interactions in school children (a group usually neglected in health surveys). Another positive aspect of this study was the high response rate of 93.7 and 81.5 and 89.05% for SES, birth weight and parental level of education respectively. In addition to BMI, this study has used WC and %BF, which provide information on body fat distribution.

## Conclusion

Concluding this study in Sub-Saharan African children shows an association between a high frequency of obesity and tall stature with high socioeconomic status. This implies that any future obesity prevention program in the North West Region of Cameroon will need to start at the family level beginning with individuals of a high socioeconomic background (especially in urban settings). However, the effect of socioeconomic background on height-obesity relationship in school age children needs further exploitation using a more accurate indicator of SES like household amenities in other regions of Cameroon and Africa.

## Competing interests

The authors declare that they have no competing interests.

## Authors’ contributions

LKN was responsible for the conception and design of the study, direct collection of data and organization, statistical analysis and drafted the manuscript. UF contributed to the conception and the design of the study as well as interpretation and analysis concepts of data. ET contributed to the design of the study, participated in data collection and contributed to drafting of the manuscript. SBD contributed to the conception and design of the study, data interpretation and drafting of the manuscript. KGP substantially contributed to the conception and design of the study, participated in data collection as well as interpretation of data. All authors revised the manuscript and gave a final approval of the submitted version.

## Pre-publication history

The pre-publication history for this paper can be accessed here:

http://www.biomedcentral.com/1471-2458/14/320/prepub
